# A task-oriented framework for dynamic generation of public health emergency plans in major infectious disease response

**DOI:** 10.3389/fpubh.2026.1831748

**Published:** 2026-10-27

**Authors:** Fangzhou Li, Ming Cong, Yue Feng, Lili Rong

**Affiliations:** 1 School of Management, Zhengzhou Shengda University, Zhengzhou, China; 2 School of Economics and Management, Dalian University of Technology, Dalian, China

**Keywords:** emergency response modeling, hierarchical task network planning, infectious disease emergency management, public health emergency planning, task-oriented emergency response

## Abstract

Public health emergency plans for major infectious diseases provide essential operational guidance for emergency response, but they suffer from high content homogeneity and limited operability, which reduces their effectiveness in real-world implementation. Although previous studies have explored task decomposition and emergency plan generation, there remains a lack of approaches that systematically extract task semantics from real emergency texts and dynamically construct executable task structures. To address this gap, this study proposes an Hierarchical Task Network(HTN)-Verb task-oriented framework for major infectious disease emergency response, in which emergency-response verbs are treated as the core carriers of task semantics to enable unified modeling of public health emergency plan texts and actual response records. The framework integrates verb-driven hierarchical task decomposition, dynamic plan generation based on practice feedback, and multi-dimensional task relationship modeling with priority-based conflict resolution. The proposed method is evaluated through a grassroots case study of Village A. Results show that emergency plans generated using the HTN-Verb framework achieve substantially higher overall effectiveness and operability than the original public health emergency plan text. The proposed approach provides a feasible pathway for transforming static emergency plans into structured and executable task networks, with practical implications for improving grassroots emergency response efficiency.

## 1 Introduction

Since the beginning of the 21st century, the world has experienced multiple major outbreaks of infectious diseases, posing severe challenges to public health management systems worldwide. Major infectious diseases are characterized by rapid transmission, wide spatial spread, long incubation periods, and strong concealment, which significantly increase the difficulty of effective response (1). Compared with natural disasters and accidental hazards, infectious disease emergencies involve greater uncertainty, leading to substantially higher response complexity (2).

Emergency plans are essential guidance for emergency responses, as they provide textual descriptions of response processes in natural language. However, in practice, public health emergency plans frequently suffer from deficiencies such as incomplete content, rigid structures, mechanical replication, and insufficient operational feasibility (3–5). Moreover, emergency plans across different administrative levels and regions exhibit a high degree of homogeneity, lacking targeted designs that reflect hierarchical or regional differences. As a result, they are often unable to effectively support the rapidly evolving response requirements associated with major infectious disease outbreaks. Therefore, developing an adaptive, more executable approach to the dynamic generation of public health emergency plans has become a critical and pressing need.

In a major infectious disease emergency response, operational actions are typically expressed in a verb-centered manner. Consequently, the types and distributions of verbs can largely reflect the task space covered by emergency plans as well as their levels of operational granularity. To better understand the gap between formal emergency plans and actual response practices, this study draws on a large corpus of emergency plan documents across multiple administrative levels, together with textual records of real-world response activities. Comparative analysis of verb usage indicates that actual response texts contain substantially richer and more fine-grained task expressions than formal emergency plans.

Furthermore, as shown in Table 1 and Figure 1, similarity analysis of verb distributions indicates that emergency plans across different administrative levels are highly similar to each other, while their similarity to actual response texts remains relatively low. These findings suggest that existing emergency plans exhibit strong homogeneity and are insufficiently aligned with real-world response practices.

**Table 1:** Cosine similarity between different texts.

Text types	Cosine similarity
Response posts vs. national-level plans	0.46
Response posts vs. provincial-level plans	0.52
Response posts vs. municipal-level plans	0.55
National-level plans vs. provincial-level plans	0.73
National-level plans vs. municipal-level plans	0.70
Provincial-level plans vs. municipal-level plans	0.83

Cosine similarity values indicate the degree of similarity between emergency plan texts at different administrative levels and actual response records.

**Figure 1:**
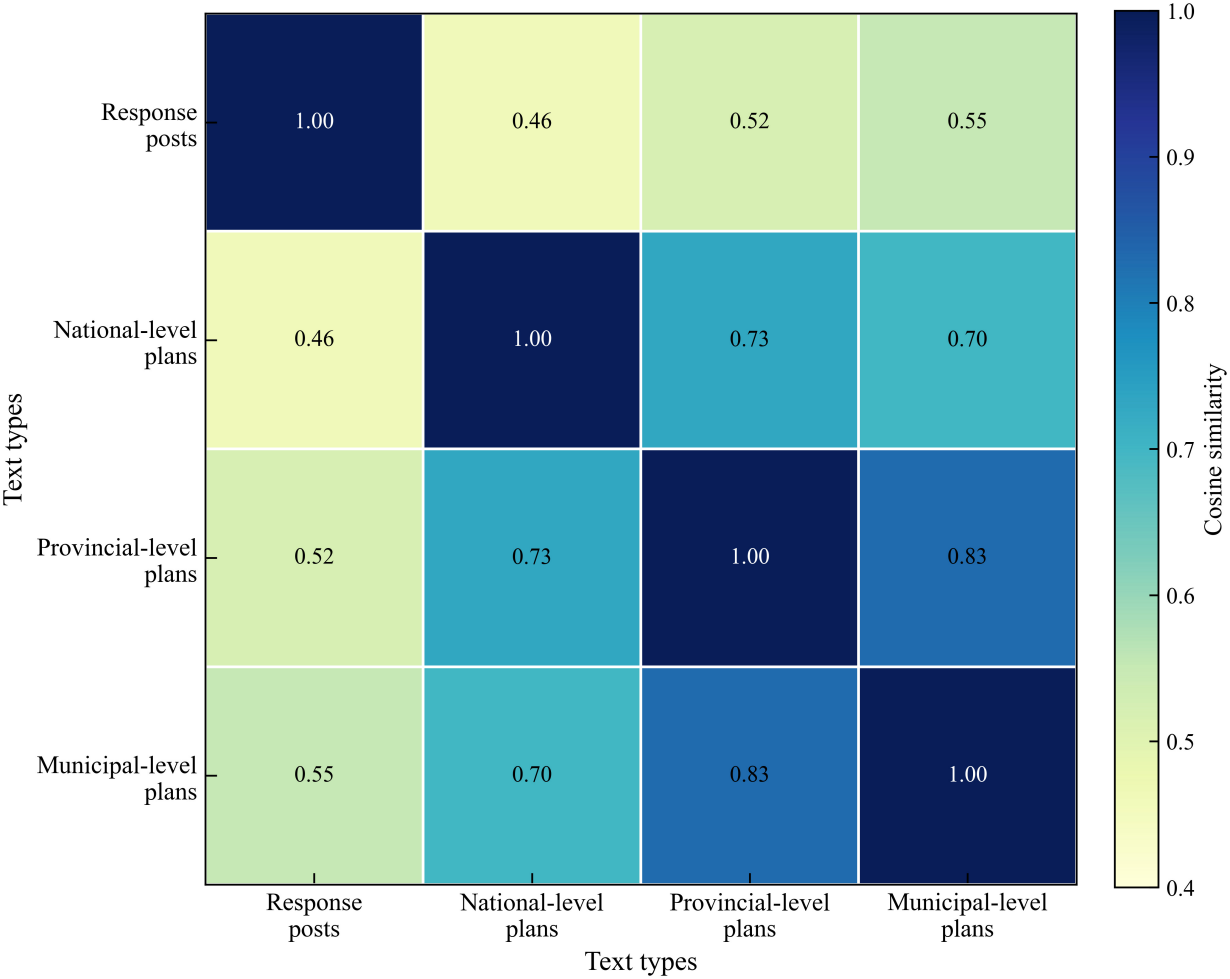
Heatmap of cosine similarity between different text types. The figure presents cosine similarity scores comparing emergency plan texts at different administrative levels with actual emergency response records.

The above findings reveal three prominent deficiencies in current emergency plans. First, the content of existing plans is insufficiently comprehensive and fails to cover the fine-grained task information required in actual emergency responses. Second, emergency plans across different administrative levels exhibit a high degree of homogeneity, with extensive textual replication and limited adjustment to level-specific or context-specific response needs. Third, a clear disconnect exists between emergency plans and real-world response practices, resulting in information gaps or inadequate task granularity that leave many concrete operational actions uncovered.

Based on these observations, this study constructs a task-oriented verb lexicon for major infectious disease emergency response. Details of the verb corpus composition are provided in Section 3.2. This verb lexicon serves as the foundational resource for subsequent task decomposition and emergency plan generation.

From a methodological perspective, existing research on task modeling and planning provides important foundations for addressing the challenges identified above. Hierarchical Task Network planning has been widely recognized as an effective approach for decomposing complex tasks into executable subtasks, offering clear advantages in incorporating domain knowledge and handling real-world planning constraints (6, 7). Foundational studies have formalized key HTN concepts, including task decomposition, ordering constraints, and applicability conditions, and have demonstrated that HTN planners often outperform classical planners in domains that require rich procedural knowledge and hierarchical structures. More recent studies in emergency management further highlight the practical value of HTN-based approaches (8). In particular, dynamic emergency decision-making models frequently integrate HTN to refine high-level response actions retrieved through case-based reasoning, enabling structured decomposition and prioritization of response tasks under evolving disaster scenarios (9). However, existing HTN applications rely almost exclusively on predefined task schemas manually crafted by domain experts. They do not address how task structures can be systematically derived from empirical emergency texts. Therefore, a clear gap remains in linking real-world emergency language, especially verb-based task expressions, to formal HTN task representations, which limits the scalability and adaptability of HTN-based approaches in practical emergency planning contexts.

Existing studies on emergency plan generation predominantly treat plans as holistic solutions to be retrieved, recommended, or selected (10). Ru et al. (11) developed a reference plan model for public health emergencies based on case-based reasoning, in which feasible plans are obtained through similarity matching with historical cases. However, emergency plans are modeled as complete case units, and their internal task structures are not explicitly represented, which prevents fine-grained adjustment of response actions. Zhang et al. (12) proposed a dynamic emergency plan generation method that applies integer linear programming to optimize response strategies under multiple scenarios and resource constraints. Moreover, the optimization target remains a set of predefined plan combinations rather than a reconfigurable structure driven by task semantics. Similarly, Zhang et al. (13) proposed the two-stage plan selection method and focused on ranking candidate plans rather than constructing new plans from underlying task behaviors. However, emergency response to major infectious diseases is characterized by long task chains, complex task dependencies, and rapidly evolving task requirements. Under such conditions, approaches that rely solely on plan-level selection or optimization are insufficient to support dynamic adjustment at the task level. Achieving highly executable emergency plans, therefore, requires the ability to identify task semantics from textual sources and to construct hierarchical task structures that can adapt to changing response demands, thereby advancing plan representation from “plan selection” toward “executable task networks.”

In existing emergency management research, relationships among tasks are often described based on process flows observed in specific cases, typically in the form of sequential, staged, or parallel execution (14). For example, Meng et al. (15) outlined urban disaster response processes in a phased manner to illustrate the basic order of task progression; however, the resulting relationship structure primarily reflects a single procedural logic. Li et al. (16) identified typical task connections, such as sequential and parallel relations, in Natech emergency analysis, but these relationships are used only to describe execution paths under specific scenarios and do not account for situations in which multiple types of relationships may coexist within the same task. Other studies emphasize the stage-based structure of task chains but continue to rely on narrative process descriptions, without abstracting the diverse logical, temporal, or resource-dependent relationships that may exist among tasks (17). As a result, when tasks are subject to multiple relational constraints, such process-oriented models often fail to reveal potential inconsistencies or restrictive interactions, and they are difficult to apply to task decomposition or emergency plan generation.

Although the above studies provide important foundations for task decomposition, emergency plan generation, and task relationship description, three critical issues remain unresolved: how to systematically extract task semantics from real-world emergency texts, how to construct task structures that can dynamically adapt to changing scenarios, and how to form executable task networks under multiple relational constraints.

To address these issues, this study makes three key contributions:

**(1) An HTN-Verb framework**. We propose an HTN-Verb decomposition framework centered on emergency response verbs, in which verbs are treated as the fundamental carriers of task semantics. By leveraging verb semantics to drive the hierarchical organization of tasks, the proposed framework enables a systematic mapping from unstructured emergency texts to interpretable task structures. This approach provides a reusable modeling paradigm for constructing hierarchical emergency task networks directly from textual data.

**(2) A dynamic public health emergency plan generation mechanism**. Building upon the HTN-Verb framework, this study develops a dynamic emergency plan generation mechanism that integrates emergency plan texts with records of real-world response practices. Through continuous incorporation of practical feedback, task structures can be progressively enriched and evolved, thereby overcoming the reliance of conventional plan generation methods on static plans and improving the adaptability of generated plans to real response demands.

**(3) Multi-dimensional task relationship modeling with priority rules**. To address complexity and potential conflicts among emergency response tasks, we introduce a multi-dimensional task relationship modeling approach that jointly considers logical, temporal, and functional relationships. In addition, priority rules based on the semantics of these relationships are incorporated to resolve potential conflicts, enhancing the consistency and executability of task networks and providing structured support for subsequent emergency plan generation and evaluation.

The remainder of this paper is organized as follows. Section 2 presents the proposed methodology. Section 3 reports the data and experimental results. Section 4 demonstrates the application and evaluation of the proposed approach. Section 5 discusses limitations and future research directions, followed by a conclusion.

## 2 Methodology

This study adopts a computational framework development and case-based evaluation design to construct and assess the proposed HTN-Verb approach. The research process consists of three interconnected phases: framework development, computational implementation and validation, and case-based application and evaluation.

In the framework development phase, emergency-response verbs are used as the core semantic units for representing and decomposing emergency tasks. The HTN-Verb framework is constructed by integrating hierarchical task decomposition, task normalization, multidimensional task relationship modeling, and a dynamic emergency plan generation mechanism.

In the computational implementation and validation phase, emergency-response texts are processed to extract task-related verbs and construct the emergency-response verb corpus. The verbs are subsequently organized according to emergency themes and hierarchical task levels through semantic representation and clustering procedures. The resulting task structures provide the computational basis for subsequent emergency plan generation.

In the case-based application and evaluation phase, the proposed framework is applied to the Village A case. Task structures and relationships derived from the original emergency plan and actual response records are used to construct the corresponding task networks, and the generated emergency plan is compared with the original plan in terms of plan adequacy and operability.

These three phases form a sequential methodological process from framework construction and computational implementation to case-based evaluation, thereby connecting the conceptual development of the HTN-Verb framework with its empirical application and assessment.

### 2.1 The HTN-Verb framework

Emergency plan formulation is a typical complex and unstructured decision-making problem. It is difficult to address using purely mathematical modeling or optimization techniques and instead relies heavily on domain knowledge in emergency management (18). HTN planning is a classical intelligent task-decomposition approach that effectively models human decision-making processes and has been widely applied in domains such as military operations, games, and emergency management (19). The core idea of HTN planning is to iteratively decompose highly complex tasks into subtasks and, ultimately, into primitive tasks that can be directly executed.

Building on the principles of HTN planning, this study proposes an HTN-Verb framework that places emergency response verbs at the center of complex task decomposition (20), as illustrated in Figure 2. The framework decomposes complex emergency tasks into clearly defined and operationally executable primitive tasks, thereby providing actionable guidance for emergency response agents. During the decomposition process, task objectives gradually evolve from vague to explicit, task content is refined from macro to micro levels, and task executability is progressively enhanced.

**Figure 2:**
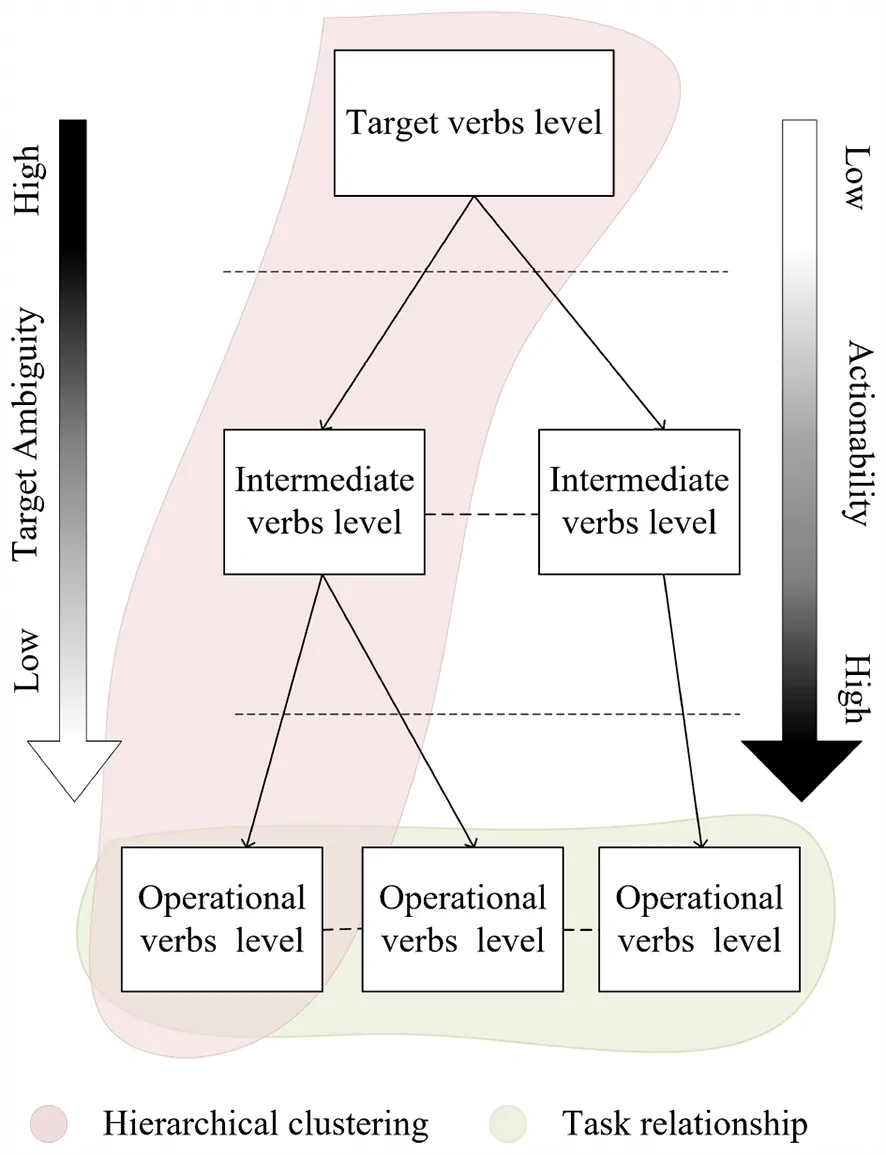
Task-oriented HTN-Verb decomposition framework. The figure illustrates the main stages of the proposed framework, including emergency verb extraction and hierarchical task decomposition.

In the area of emergency management, emergency themes represent abstractions of emergency tasks and can be regarded as macro-level complex tasks (21). Correspondingly, **target verbs** are associated with complex tasks and represent strategic objectives or desired end states of emergency response. These verbs typically provide high-level guidance for the entire task framework and are characterized by abstraction, generality, and ambiguity. They are usually articulated by top-level decision-making authorities (e.g., central governments) to guide lower-level actors, such as *control, recover*, or *response*.

At the lowest level, **operational verbs** correspond to primitive tasks that can be directly executed. These verbs represent the most concrete and actionable tasks, closely aligned with the actual behaviors of executing agents. They are characterized by urgency, measurability, and direct operability, and are typically carried out by local or grassroots actors responsible for on-site implementation and real-time feedback, such as *sample* or *inject*.

Between these two levels, **intermediate verbs** correspond to subtasks and serve a coordinating role that bridges strategic objectives and concrete operations. They transform abstract goals into partially operational tasks and are characterized by connectivity and partial executability. Their execution is commonly associated with mid-level administrative entities, such as provincial or municipal authorities, and includes actions such as *organize* or *schedule*.

In terms of verb semantic representation and classification, mainstream approaches include BERT-based pretrained language models and traditional word embedding models (22, 23). Comparative studies have shown that BERT-based models, by leveraging contextualized representations, can more accurately capture fine-grained semantic differences between verbs, whereas traditional word embedding models rely primarily on lexical similarity and lack deep contextual understanding (24). On this basis, this study performs hierarchical clustering of emergency response verbs to align with the HTN-Verb framework.

Specifically, the bert-base-chinese model is employed to vectorize emergency response verbs (23). An average pooling strategy is applied to generate fixed-dimensional embeddings for each input text, capturing deep semantic features. Principal Component Analysis (PCA) is then used to reduce the dimensionality of the high-dimensional embeddings while retaining the principal variance components. Finally, Agglomerative Clustering is applied to group emergency verbs under the constraints of the HTN-Verb framework, revealing their structural relationships and category distributions in semantic space (25). This approach enables intelligent extraction and decomposition of task verbs from emergency texts, enhances the structural organization and dynamic adaptability of the verb lexicon, and provides a practical foundation for subsequent emergency plan generation.

### 2.2 Task normalization model

According to Ni et al. (26), an emergency task consists of three core elements: action, agent, and resource, which correspond to how the task is performed, who performs it, and what is used to perform it, respectively. Among these elements, the action component represents the core of a task and can be aligned with agent responsibilities and task objectives, as illustrated in Figure 3. Based on this perspective, an emergency task can be formally represented as a triplet, as shown in Equation 1:


$$\begin{array}{l} \text{Task}=\left(T_{\text{action}},T_{\text{agent}},T_{\text{resource}}\right) \end{array}$$ (1)

**Figure 3:**
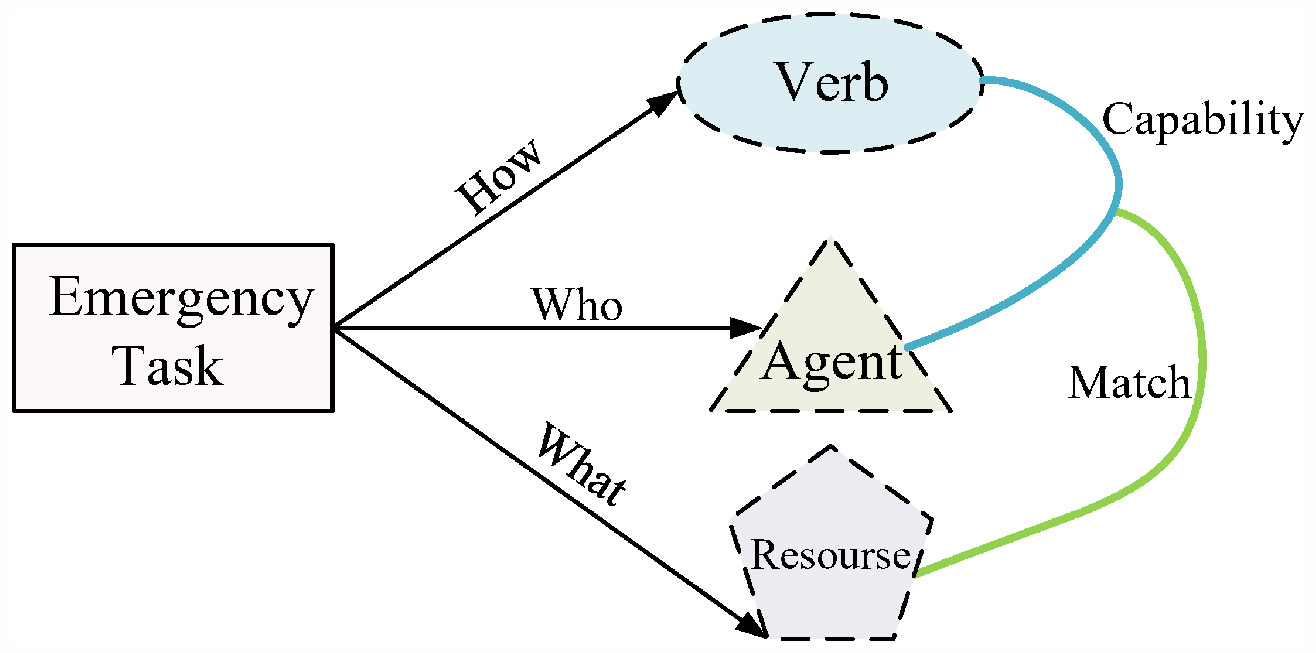
Relationship map of emergency response task elements. This diagram illustrates the three primary components of the task and the matching relationships between them.

For example, the task of transporting masks can be represented as shown in Equation 2:


$$\begin{array}{l} \langle \text{transport},\text{volunteers},\text{transport vehicle}\rangle . \end{array}$$ (2)

Within the task normalization model, resource elements are associated with task execution and can be understood along three aspects: resource types, resource constraints, and allocation relationships. Resource types refer to categories such as personnel, equipment, materials, and information required to support specific tasks. Resource constraints reflect practical conditions, including availability, capacity, and temporal requirements. Allocation relationships describe how resources are linked to particular tasks, indicating which resources are required for specific actions under the responsibility of corresponding agents. This representation provides a structural basis for incorporating resource elements into the task modeling framework.

In practical emergency response settings, once tasks are specified, the corresponding resource types can generally be identified. For example, tasks such as “conduct testing” or “implement isolation” imply associated resources, including testing kits, medical personnel, and isolation facilities. Within the task normalization model, resources are associated with specific verb-agent pairs, forming an extended representation of (agent, verb, and resource). Resource types are inferred from the semantic meaning of actions together with their responsible agents, reflecting the supporting conditions required for task execution. Consistent with the verb-centered design of this study, the current analysis focuses primarily on actions and agents, while resource-related aspects are not explicitly modeled in detail. However, due to data limitations and the scope of this study, resource modeling in the current framework remains at a conceptual level. It is not explicitly developed into a structured or quantitative form. In particular, resource-related factors such as availability, capacity, and temporal constraints are not incorporated into the current model. These aspects will be further considered in future work to enhance the completeness of the task modeling framework.

Emergency verbs do not exhibit a one-to-one correspondence with emergency tasks. Instead, the mapping between verbs and tasks is jointly influenced by multiple factors, including executing agents, emergency themes, and available resources, resulting in a multidimensional and dynamic mapping relationship. The same emergency verb may convey substantially different task semantics under different executing agents. For instance, “*assessment*” combined with “*hospital*” typically refers to physicians' clinical evaluation of patients' conditions, whereas “*assessment*” combined with “*municipal health commission*” more often denotes a comprehensive evaluation of epidemic trends at the administrative level. This observation indicates that relying solely on surface verb meanings or semantic similarity is insufficient to stably characterize task semantics. The fundamental reason lies in the fact that verb interpretations are constrained by responsibility boundaries under different emergency themes.

Therefore, it is necessary to categorize and constrain emergency verbs by grounding them in the division of responsibilities within the emergency management system, integrating agent functional roles with emergency themes (27). To this end, this study systematically reviews public health emergency documents at different administrative levels and performs identification and normalization of emergency response agents within the texts. Through this process, the agent element of emergency tasks (i.e., *who performs the task*) is explicitly specified, with partial results presented in Table 2. This provides an operational foundation for subsequently constraining emergency verbs to concrete task semantics and associating them with required resources.

**Table 2:** Major infectious disease emergency response agents (partial list).

Emergency response agents Prefixes	
Universities	Central Committee of the Communist Party
Research institutions	State Council
Enterprises	National Health Commission
Civil affairs authorities	Public Security
Ministry of Finance	Medical and health institutions
Audit authorities	Tourism authorities
Armed Police	Emergency Command Department
Fire services	Centers for Disease Control and Prevention
Water resources authorities	Immigration and border inspection
Energy sector	Local government
Housing and urban development authorities	Transportation authorities
Big data authorities	Ministry of Science and Technology
Subdistrict offices	…

This table lists several representative emergency response agent prefixes. The ellipsis (…) indicates that additional entries are not shown.

In addition, this study identifies 12 major public health emergency themes and their corresponding primary responsible departments in Table 3. These themes serve as a reference framework for emergency verb semantic mapping and task normalization, enabling consistent and interpretable task representations across different response contexts.

**Table 3:** Correspondence between emergency themes and their primary responsible departments.

Emergency theme	Primary responsible department
Material supply	Ministry of Industry and Information Technology
Public communication and education	Publicity Department
Medical treatment and rescue	Medical institutions
Environmental protection	Department of Ecology and Environment
Financial support	Ministry of Finance
Transportation and logistics	Transportation authorities
Information reporting	National Health Commission
Technical support	Ministry of Science and Technology
Legal regulation	Ministry of Justice
Emergency command	Emergency Command Center
Control and supervision	Centers for Disease Control and Prevention
Supervision and evaluation	Local governments

The table summarizes major emergency themes in public health emergencies and the corresponding departments primarily responsible for each theme.

### 2.3 Multi-dimensional task relations description

In task decomposition and simulation, a standardized description of task relationships forms the foundation for constructing executable task networks (28). Emergency response tasks typically exhibit deep hierarchies and complex dependencies. Vertically, tasks are organized into hierarchical structures consisting of complex tasks, subtasks, and primitive task (29). Horizontally, tasks may form networked structures in which multiple types of relationships coexist, such as logical, temporal, and functional relations. Accordingly, clearly identifying and categorizing different types of task relationships is a critical step in ensuring the rationality of task decomposition and the effectiveness of plan generation.

Existing studies generally classify task relationships into several categories, including logical, temporal, and functional relations (30). To facilitate subsequent modeling, this study focuses on these three core relationship types, which are described below.

**(1) Logical relations** characterize conditional dependencies among primitive tasks and typically include three structures: AND, OR, and NOT.

1. AND relation: A subsequent task can be initiated only after all prerequisite tasks have been completed. For example, “construct temporary facilities” can be executed only after both “site leveling” and “material transportation” are completed.
2. OR relation: Completion of any one prerequisite task is sufficient to trigger the subsequent task. For instance, either “restoring hospital power supply” or “activating backup generators” can enable “ensuring hospital electricity supply.”
3. NOT relation: The non-completion of a task constitutes the triggering condition for a subsequent task. For example, when an area has not been assessed as being in a normal state, “control measures” need to be implemented.

**(2) Temporal relations** are used to describe the precedence or simultaneity of tasks along the time dimension and constitute the basis for arranging emergency actions in the temporal dimension. This type of relation does not involve resource dependencies or functional associations; rather, it solely reflects the temporal ordering of task execution, such as “screening before isolation” or “conducting nucleic acid testing and information registration simultaneously.”

**(3) Functional relations** capture the functional interactions among tasks and include three categories: dependency, independence, and coupling.

1. Dependency relation: Task B requires resources, information, or states provided by Task A, although the two tasks do not need to be executed consecutively. For example, “hospital treatment” depends on information regarding the “patient's disease status.”
2. Independence relation: Tasks do not directly affect each other and can be executed independently or in parallel, such as “setting up traffic checkpoints” and “conducting medical treatment.”
3. Coupling relation: Tasks mutually influence each other and depend on each other's outputs. For instance, “infectious disease surveillance” and “epidemiological investigation” are mutually supportive: surveillance data determine the scope of investigation, while investigation results in turn enrich surveillance information.

Through the definition of these relationship types, multi-dimensional interdependencies among emergency response tasks can be systematically characterized, providing a solid foundation for constructing task networks with clear structure and rigorous logic.

Among the various types of task relations identified in existing studies, logical relations, temporal relations, and functional relations are considered in this study to capture most of the critical interdependencies among emergency response tasks and to serve as the most representative relation types for task network construction. Other commonly used relation types can often be expressed as combinations of these three, for example, process relations may be decomposed into temporal relations combined with logical conditions, while resource relations may be interpreted as temporal constraints coupled with functional dependencies. Based on this modeling perspective, this study adopts the above three relation types to characterize the horizontal associations among emergency tasks and constructs corresponding task relationship matrices, providing a structured foundation for subsequent task network reasoning and plan generation.

When constructing task relationship matrices, a common practice is to build separate matrices for individual relation types independently (31). However, when multiple relation types are integrated into a single task network, inconsistencies or even conflicts may arise. For instance, a temporal relation may require Task A to precede Task B, while a logical relation may allow Task B to act as a triggering condition that feeds back into Task A. Similarly, a functional relation may designate Task C as a critical dependency, whereas the logical relation matrix lacks the corresponding conditional constraints. Such cross-relation contradictions are relatively common in multi-dimensional relationship modeling (32). Without a systematic conflict resolution mechanism, it is difficult to ensure the overall consistency and executability of the task network.

From the perspectives of task semantics and emergency response objectives, the three types of relations play distinct roles within a task network. Functional relationships characterize the actual contribution of each task to overall emergency objectives. If critical functional dependencies are disrupted, effective response outcomes cannot be guaranteed, even when tasks are executed on time and remain logically consistent. Consequently, functional relations are of the highest importance when determining whether a task is necessary and meaningful to the overall response.

Temporal relations primarily constrain the execution order of tasks and are directly associated with response efficiency and the feasibility of resource scheduling. However, under conditions where a certain degree of redundancy and scheduling flexibility is available, temporal constraints can often be locally adjusted through modifications of time windows or parallel execution arrangements.

In contrast, logical relations reflect triggering conditions and dependency paths among tasks. Provided that key functional dependencies and core temporal constraints are preserved, logical relations can often be restructured by adjusting execution sequences or by relaxing or tightening triggering conditions. As a result, logical relations generally exhibit a higher degree of flexibility and adjustability.

Based on the semantic characteristics of the three relationship types and their respective roles in emergency response, this study adopts the following conflict-resolution priority for the proposed framework: **functional relations** **>** **temporal relations** **>** **logical relations**. This ordering is used as a modeling rule to preserve critical functional dependencies while coordinating temporal arrangements and logical conditions when multiple relational constraints coexist.

For implementation, the identified operational-level tasks were represented using three separate relationship matrices corresponding to logical, temporal, and functional relations. Each task pair was examined according to the definitions above and encoded in the corresponding matrix. Logical relations were represented as AND, OR, NOT, or no relation; temporal relations as precedence, simultaneity, or no relation; and functional relations as dependency, coupling, or independence. Where multiple relationship constraints produced inconsistent task-network structures, the predefined priority rule was applied to resolve the conflict before constructing the final task network.

For example, when a functional dependency between two tasks conflicts with a temporal or logical constraint, the functional dependency is retained first, while the temporal arrangement or logical triggering condition is adjusted where possible to maintain network consistency.

### 2.4 Dynamic emergency plan generation mechanism

In emergency management systems, the generation of emergency plans does not rely solely on static emergency plan documents; it also requires the continuous incorporation of operational experience and task knowledge accumulated during actual response processes (33). In this situation, this study develops a dynamic emergency plan generation mechanism centered on emergency response verbs. Through text mining of existing emergency plan documents and real-world response records, emergency verbs with clear action orientations are extracted and semantically constrained according to the responsibility boundaries of emergency response agents. This process clarifies task semantics and responsible agents, while establishing the necessary semantic foundation for subsequent task-resource allocation.

On this basis, emergency plans oriented toward practical response demands are generated to support the continuous progression of emergency response activities. As the response unfolds, the system can identify newly emerging tasks that are not covered by existing plans and record and organize the associated task information. These newly identified tasks are incorporated into updated response records, further enriching the emergency verb lexicon. In this way, an evolutionary closed-loop mechanism, comprising plan generation, response execution, task augmentation, and knowledge updating, is formed in Figure 4, thereby enhancing the adaptability of emergency plans to dynamic response requirements.

**Figure 4:**
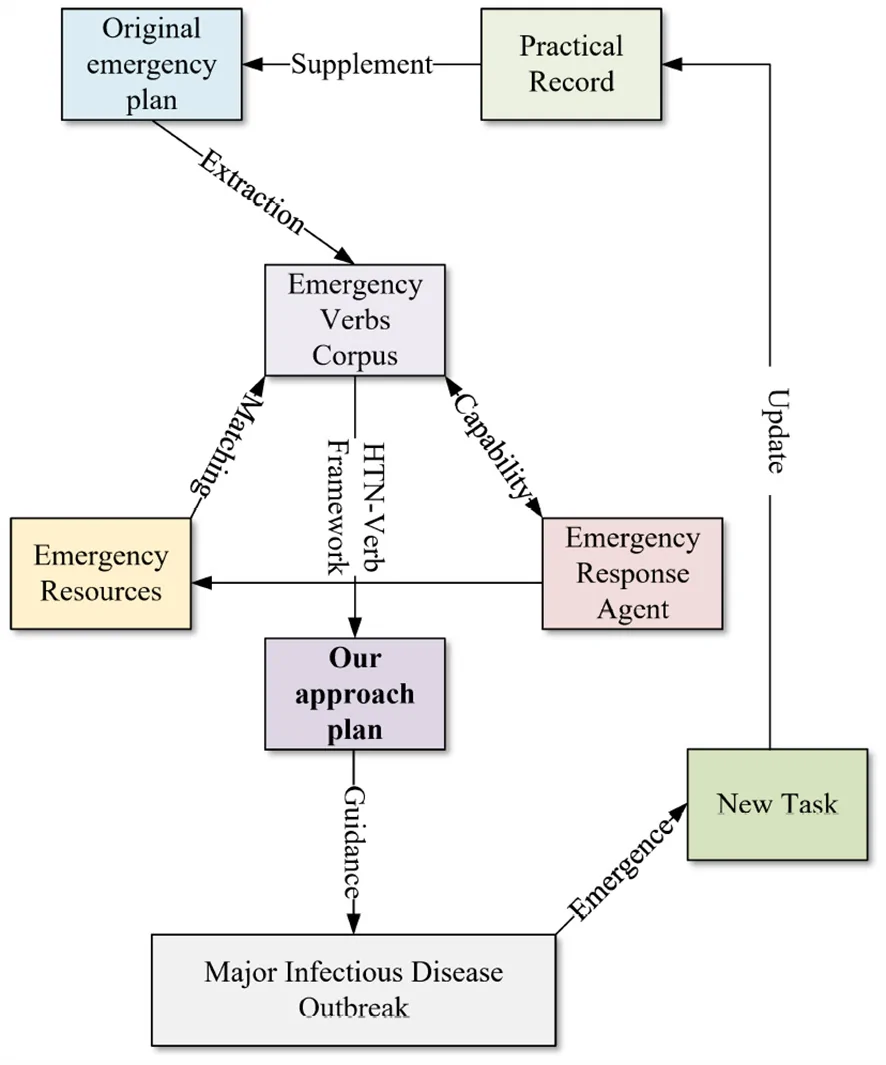
Schematic diagram of the methodology for dynamic generation of emergency response plans for major infectious diseases. This diagram illustrates the dynamic generation process of major infectious disease emergency response plans. The light blue box in the upper left corner represents the initial state. Following the sequential downward flow, the process proceeds to the gray section at the bottom before feeding back to the light blue section in the upper left corner, completing one dynamic update cycle.

## 3 Data and analysis

### 3.1 Data sources

Emergency plans, laws, regulations, and other emergency-related documents concerning public health emergencies were systematically collected across multiple administrative levels, resulting in a total of 108 documents spanning the national, provincial, and municipal levels. All collected documents are written in Chinese, reflecting the linguistic characteristics of public health emergency management in China. A purposive, criterion-based corpus construction strategy was adopted to ensure the relevance and informational completeness of the collected documents. Documents were retained if they contained relatively complete descriptions of public health emergency response tasks and plan structures. Documents with substantially incomplete content or structures were excluded; for example, plans covering only a limited subset of emergency themes (e.g., four themes) or lacking major structural components were not included in the corpus.

To compensate for potential omissions of operational-level tasks in formal emergency plans, this study further collected textual records generated during actual public health emergency response processes, which are likewise Chinese-language texts. Specifically, using keywords such as “infectious disease” and “emergency response” or “epidemic” and “emergency response”, posts published by official accounts on the Weibo platform were crawled for the period from January 2020 to December 2022 and used as supplementary textual sources to the emergency documents. The keyword combinations were selected to balance domain specificity and coverage: “infectious disease” directly corresponds to the target emergency type, whereas “epidemic” captures commonly used expressions in response records; both were combined with “emergency response” to focus retrieval on response-related content. Retrieved posts were retained only when they were directly related to major infectious disease emergency response and contained substantive task information. Advertisements, reposts, duplicate records, and posts without substantive emergency-response task information were excluded. When identical content was published repeatedly, only one record was retained. These posts were all written in Chinese, ensuring linguistic consistency with the emergency plan corpus. Weibo was selected as a supplementary source because official government and public-health accounts use the platform to publicly disseminate timely information on emergency-response activities, providing observable records of operational activities that complement the normative task descriptions contained in formal emergency documents. The period covers different stages of epidemic response, from the initial outbreak through subsequent prevention and control phases, thereby capturing response records generated under different stages of emergency management.

The two data sources therefore serve complementary purposes: formal emergency documents provide normative and institutionally defined response tasks, whereas official Weibo posts provide supplementary records of publicly reported operational activities. The latter are not assumed to provide a complete representation of real-world emergency response practices. Moreover, restricting the data sources to officially released Chinese-language texts helps ensure the authority and consistency of the information while reducing noise introduced by individual or informal expressions. All data is derived from publicly available sources. The data collection and analysis complied with the terms and conditions of the respective platforms and data providers.

### 3.2 Data processing

This section describes the detailed process of verb extraction and semantic analysis. The data processing pipeline, as shown in Figure 5, consists of three main steps: text preprocessing, verb extraction, and semantic representation.

**Figure 5:**
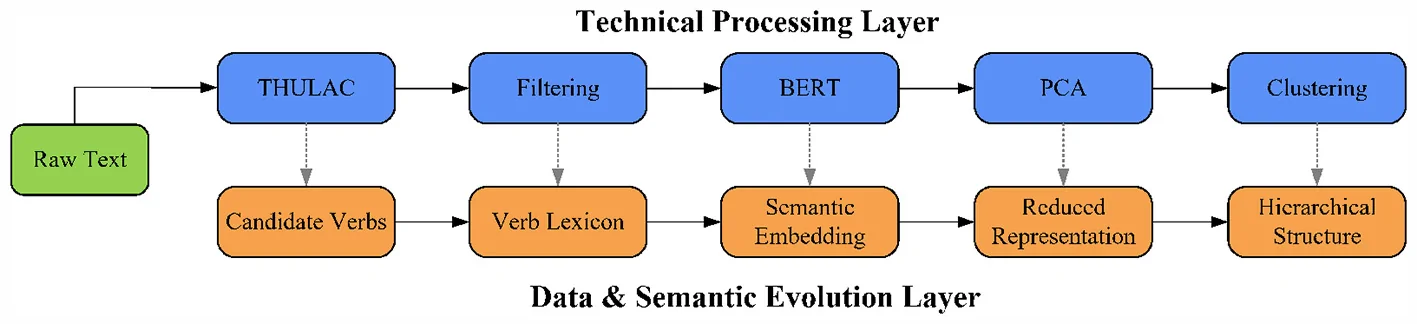
Dual-layer NLP pipeline for verb extraction and hierarchical structure construction. The upper layer shows the processing methods, and the lower layer depicts the transformation of data into hierarchical task structures.

First, the raw texts are cleaned to reduce noise and standardize the corpus. Noisy elements, including special characters (e.g., “[ ]” and “@”), dates, English tokens, and URLs, are removed, and punctuation is standardized. The cleaned Chinese texts are processed using the THULAC toolkit (34) for word segmentation and part-of-speech tagging. Verbs are extracted from the corpus based on part-of-speech annotations. To ensure relevance to emergency response tasks, only verbs with clear operational meaning are retained, while general or non-task-related verbs are removed. In addition, only two-character verbs with a frequency of no less than five are preserved to improve robustness and reduce noise. To further enhance reliability, the automatically extracted verbs were manually reviewed to remove items without clear task-oriented or emergency-response meanings. After preprocessing and manual screening, a total of 579 verbs were identified from response records, while 177, 274, and 293 verbs were extracted from national-, provincial-, and municipal-level emergency plans, respectively. After filtering and integration, a task-oriented emergency verb lexicon containing 976 verbs was constructed. The lexicon is publicly available at: https://github.com/konglong12315/HTN-Verb.

Second, the extracted verbs are encoded into semantic vector representations using a pre-trained BERT model (bert-base-Chinese) (35) implemented with the Transformers library. For each input, the last hidden-state representations generated by BERT were averaged across tokens using mean pooling to obtain a fixed semantic embedding.

Finally, PCA was subsequently used to reduce the embeddings to two dimensions (n_components = 2, random_state = 42). The reduced representations were clustered using Agglomerative Clustering with Euclidean distance and Ward linkage. The number of clusters was set to three, corresponding to the three task levels defined in the HTN-Verb framework: target, intermediate, and operational levels. To assess the quality of the resulting clusters, the silhouette coefficient was calculated for each of the 12 emergency themes. Across the 12 emergency themes, the mean silhouette coefficient was 0.528, with theme-specific values ranging from 0.490 to 0.544, indicating reasonable within-cluster cohesion and between-cluster separation across the emergency themes. Detailed implementation settings and software configurations are provided in the Supplementary Table S1.

### 3.3 Characteristics of the verb corpus

Based on the semantic representations and clustering results obtained in Section 3.2, a three-level hierarchy of emergency-response verbs is constructed. This hierarchical structure is interpreted within the HTN-Verb framework, where verbs are categorized into target-level, intermediate-level, and operational-level tasks.

Because emergency-response verbs are often polysemous or cross-thematic, their task meanings cannot always be determined from the verb form alone. The same verb may express different emergency functions depending on its textual context, associated agent, operational object, and response objective. Consequently, the mapping between verbs and emergency themes is not necessarily one-to-one, as discussed in Section 2.2. To reduce ambiguity in verb interpretation, thematic assignment was therefore based not only on lexical semantics but also on contextual usage, operational function, domain-specific collocations, and goal orientation.

To constrain task interpretation, we therefore assign verbs to 12 emergency themes using the following five rule-based criteria:

**Rule 1 (Semantic relevance)**. If the semantic meaning of a verb is relevant to a theme, matching is performed using synonyms or near-synonyms. For example, “treat” can be matched to the “medical treatment and rescue” theme.

**Rule 2 (Contextual usage)**. When a verb is used in a specific context, its association with a particular theme may become stronger. For instance, “isolate” can be associated with the “control and supervision” theme.

**Rule 3 (Operational function)**. Verbs are matched to themes according to their functional roles in concrete operations. For example, “allocate” can be mapped to the “material supply” theme.

**Rule 4 (Domain collocations)**. Common collocations and phrases used within the domain are referenced to determine verb-theme associations. For example, in public health contexts, “sample” is commonly associated with the “control and supervision” theme.

**Rule 5 (Goal orientation)**. Verb usage should contribute to achieving a specific objective and is therefore mapped to the corresponding theme. For example, the verb “audit” can be assigned to the “financial support” theme because its purpose is to ensure compliance in the use of funds.

To reduce subjectivity in the thematic assignment, an inter-rater validation procedure was conducted before the full-scale classification. Two annotators independently assigned a pilot sample of 50 verbs to the predefined emergency themes according to the above criteria. The independent annotations agreed on 41 of the 50 instances, corresponding to an observed agreement of 82.0%. Inter-rater reliability was further assessed using Cohen's kappa, yielding κ = 0.806. Disagreements were subsequently reviewed and resolved through discussion. Following the pilot consistency check, the remaining thematic assignments were cross-checked, and disputed cases were reviewed through discussion until consensus was reached.

These criteria are designed to reflect semantic relevance, contextual usage, operational function, domain collocations, and goal orientation. Based on this rule-based assignment, verbs are organized into theme-specific clusters, as illustrated in Figure 6.

**Figure 6:**
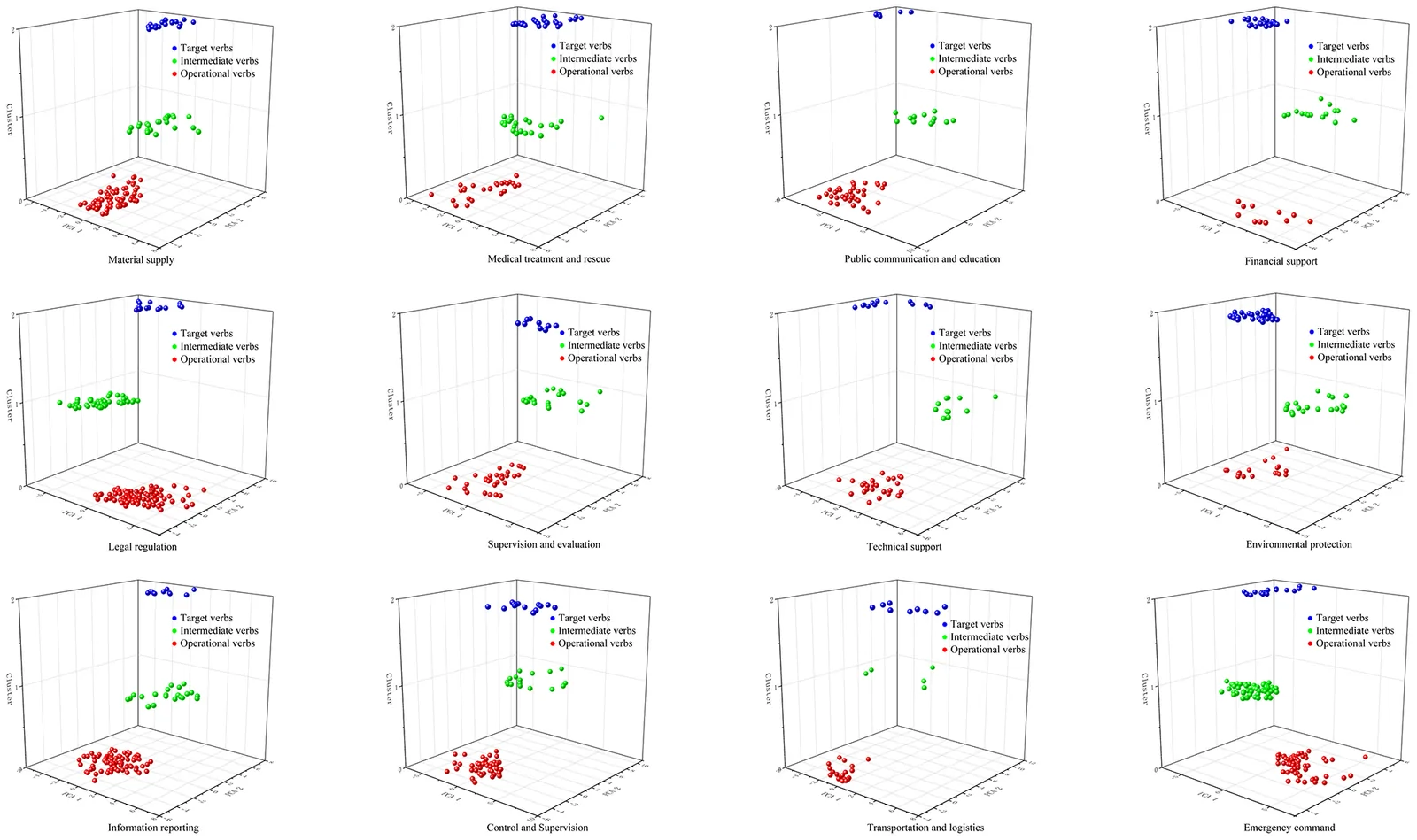
Verb hierarchical clustering results for each emergency theme. The figure presents the hierarchical clustering of emergency-response verbs under different emergency themes, showing the distribution of goal-level, intermediate-level, and operational-level verbs in the semantic embedding space.

From the perspective of the total number of emergency-response verbs associated with each emergency theme, Emergency command, Information reporting, and Legal regulation contain relatively larger verb inventories, whereas Transportation and logistics and Technical support exhibit comparatively fewer verbs. This disparity reflects an imbalance in the richness of task expression across different emergency themes and provides a basis for further analysis of semantic emphasis at different task levels within each theme.

Examining the distribution of emergency-response verbs across the target, intermediate, and operational levels reveals clear differences in representational focus among emergency themes. In terms of absolute counts, themes such as Information reporting, Legal regulation, and Material supply include relatively more target-level verbs, whereas intermediate-level verbs are generally fewer in number and more dispersed across themes. By contrast, operational-level verbs are more prevalent in themes such as Environmental protection and Financial support, indicating a stronger concentration of task expressions at the execution level in these themes.

Building on these distributional characteristics, the internal task configurations of different emergency themes are further analyzed from the perspective of hierarchical proportions in the Figure 7. The results show that, in most emergency themes, target-level verbs account for more than 50% of the total, with particularly high proportions observed in Information reporting, Public communication and education, and Material supply. This pattern suggests that task design within these themes places greater emphasis on macro-level goal formulation and normative guidance. In contrast, the proportion of intermediate-level verbs remains consistently low across themes and lacks a stable distribution pattern, indicating that intermediate-level tasks have not yet formed a clearly defined and independent structural role within the current task system. This hierarchical configuration weakens the continuity between task objectives and concrete operations, resulting in a structure in which the target and operational levels are relatively prominent, while the intermediate level is comparatively underrepresented. Meanwhile, operational-level verbs account for a higher proportion in professionally specialized themes such as Medical treatment and rescue and Environmental protection, highlighting their stronger reliance on concrete execution behaviors.

**Figure 7:**
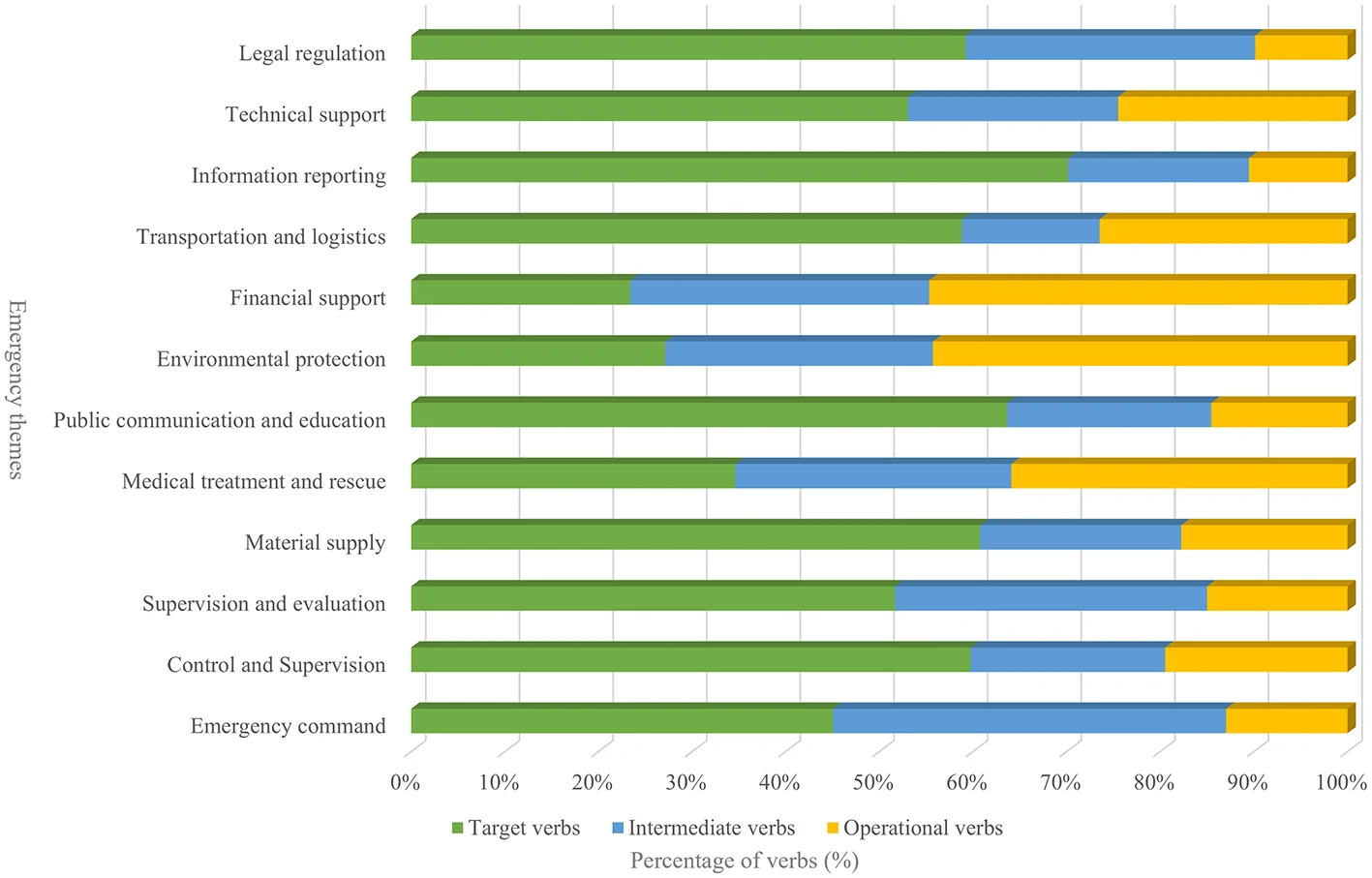
Percentage distribution of emergency verbs across emergency themes. The figure shows the proportion of target-level, intermediate-level, and operational-level verbs within each emergency theme, illustrating differences in task-level composition across themes.

Overall, the distribution of emergency-response verbs across different themes and hierarchical levels reveals a clear hierarchical configuration bias in the current emergency task system. Target-level task expressions tend to be relatively concentrated, operational-level tasks assume primary execution responsibilities in action-oriented themes, whereas the functional role of the intermediate level remains less pronounced, indicating substantial room for further optimization and strengthening.

### 3.4 Case scenario: Village A

Village A is a typical administrative village with approximately 500 households. The village is equipped with a rural cooperative medical clinic staffed by two village doctors. Since January 2020, epidemic prevention and control measures have been implemented under the unified guidance of the township government. While Village A possesses basic public health service capacity, it relies heavily on township-level and county-level authorities for medical expertise, emergency supplies, and financial support, reflecting the characteristic constraints of emergency resources at the grassroots level.

Village A was purposively selected as the case study because it provides both a formal emergency plan and records of actual epidemic-response activities, allowing the task structures specified in the plan to be compared with those observed during implementation. In addition, the case involves several characteristics directly relevant to the proposed HTN-Verb framework, including limited grassroots response resources, cross-level governmental coordination, multi-agent participation, dynamic task adjustment, and continuous resource input. These characteristics make the case particularly suitable for examining whether the proposed framework can identify and organize operational tasks that are insufficiently represented in the formal plan. The case was therefore selected for its analytical suitability rather than to claim to be statistically representative of all grassroots emergency-response settings.

The public health emergency plan implemented in Village A was formulated by the township government and adopts an organizational, responsibility-oriented structure. It is organized around themes such as emergency command, medical treatment and rescue, transportation and logistics, public communication and education, financial support, and environmental protection, with an emphasis on defining the responsibilities of different emergency-response agents.

By contrast, during the actual epidemic response, prevention and control activities were jointly organized by township- and village-level officials and dynamically carried out with support from county-level departments. Members of the village committee, township officials, volunteers, and medical personnel participated collectively in epidemic control. Specific tasks included population screening, checkpoint duty, nucleic acid testing, vaccination, quarantine management, etc. These tasks were continuously adjusted in response to epidemic developments, while resources were replenished through public funding and social donations. Overall, the actual response exhibited characteristics of multi-agent collaboration, dynamic task generation, and cross-level resource allocation, which differ markedly from the relatively static and responsibility-driven structure of the formal emergency plan.

## 4 Application and evaluation

### 4.1 Implementation of the HTN-Verb framework to Village A

Based on the constructed emergency task verb lexicon, the emergency plan text implemented in Village A and the textual descriptions of its actual epidemic response process were analyzed as two distinct corpora. Emergency-response verbs were extracted from both corpora and classified by theme and hierarchical level. Selected statistics are reported in Table 4. The results indicate that the emergency plan text covers seven emergency themes and contains 98 emergency-response verbs, among which 18 operational-level verbs correspond to 12 emergency-response agents. In contrast, the actual response texts involve 12 emergency themes and 201 emergency-response verbs, including 63 operational-level verbs associated with 21 emergency-response agents. These differences show that the actual response process substantially exceeds the formal plan in terms of theme coverage, task granularity, and the number of participating agents.

**Table 4:** Partial verb recognition results from two types of emergency texts for major infectious diseases in Village A.

Text type	Emergency verb	Emergency theme	Task level
Emergency plan text	respond	Emergency command	Target level
Emergency plan text	organize	Emergency command	Target level
Emergency plan text	command	Emergency command	Intermediate level
Emergency plan text	prevent and control	Control and supervision	Intermediate level
Emergency plan text	eliminate	Environmental protection	Operational level
Emergency plan text	sample	Medical treatment and rescue	Operational level
…			
Actual response text	respond	Emergency command	Target level
Actual response text	terminate	Emergency command	Target level
Actual response text	ensure	Material supply	Target level
Actual response text	receive	Material supply	Operational level
Actual response text	disseminate	Public communication and education	Operational level
Actual response text	verify	Supervision and evaluation	Operational level
…			

The table presents representative examples of emergency-response verbs identified from emergency plan texts and actual response records, together with their corresponding themes and task levels. The ellipsis (…) indicates that additional entries are not shown.

To analyze relationships among operational-level tasks, task relationship matrices were constructed for both the emergency plan and the actual response texts following the approach in Section 2.3. The results show that only under Control and supervision, Information reporting, and Medical treatment and rescue do operational-level tasks in the plan form relatively clear relational structures, whereas task relationships under other themes are sparsely specified.

By contrast, using operational-level verbs extracted from the actual response texts, task relationships were systematically identified across all 12 emergency themes. When relationships could not be directly inferred from textual semantics, word-embedding clustering results were used as auxiliary evidence. This process yielded a comprehensive operational-level task relationship matrix for Village A, based on which executable emergency response plans were generated.

In this application, the relationship matrices were used to organize the identified operational-level tasks into the final task network. The subsequent comparison between the generated plan and the original emergency plan therefore provides a case-based assessment of the resulting task-network structure and plan operability. However, because no independently annotated ground-truth relationship set was available, this assessment should not be interpreted as a direct validation of individual relationship labels.

### 4.2 Evaluation metrics and indicator

The effectiveness of emergency plans is evaluated from two dimensions: plan adequacy and plan operability (36). Plan adequacy focuses on the completeness and coverage of plan content and is assessed using four indicators: emergency themes, executing agents, emergency-response verbs, and emergency resources. Plan operability emphasizes the feasibility of task organization and execution and is evaluated using two indicators: task workflow and operational feasibility, as shown in Figure 8. The description of the parameters for this subsection is detailed in Table 5.

**Figure 8:**
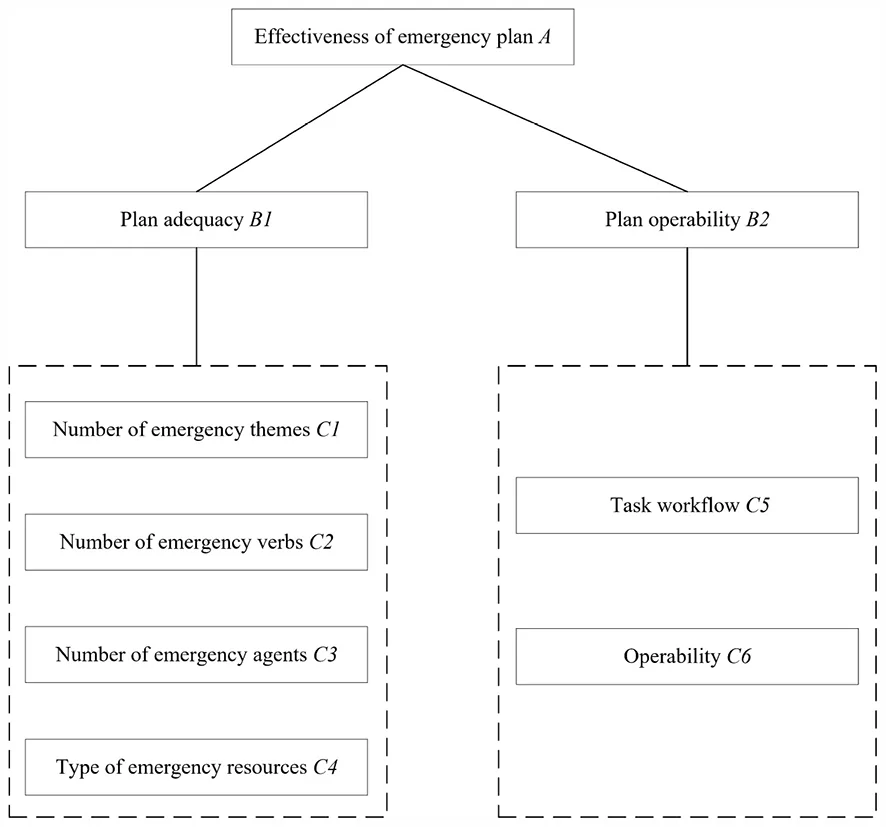
Emergency response plan effectiveness evaluation indicators. The figure illustrates the hierarchical structure of the evaluation indicators, including plan adequacy and plan operability, and their associated secondary indicators used to assess emergency plan effectiveness.

**Table 5:** Evaluation metrics and indicators used in Section 4.2.

Symbol	Description	Location
*N*	Number of operational-level tasks	Equations 5, 6
*k*_*i*_	Number of reachable tasks from task *i*	Equations 5, 6
*R*	Task workflow indicator (C5)	Equation 6
*O*	Operational feasibility indicator (C6)	Equation 7
*V*_*i, j*_	Number of verbs at task level *j* under emergency theme *i*	Equation 7
*A*	Overall effectiveness of emergency plan (top-level objective)	Table 6
*B*1	Plan adequacy (first-level criterion)	Table 6
*B*2	Plan operability (first-level criterion)	Table 6
*C*1–*C*4	Number of emergency themes, verbs, agents, and resource types	Table 6
*C*5	Task workflow (quantified by *R*)	Table 6
*C*6	Operational feasibility (quantified by *O*)	Table 6
*CR*	Consistency ratio of the judgment matrix (acceptable if *CR* < 0.1)	Table 5

The table summarizes the key evaluation metrics, parameters, and AHP-based indicators used to assess emergency plan effectiveness. These symbols and indicators correspond to the equations and tables presented in Section 4.2.

The indicator weights were determined using the Analytic Hierarchy Process (AHP), with the weights shown in Table 6. The pairwise comparison judgments used in the AHP were provided by domain expert with research experience in emergency planning. The expert assessed the relative importance of indicators at each hierarchical level using the conventional 1–9 pairwise comparison scale, where 1 indicates equal importance and 3, 5, 7, and 9 indicate progressively stronger importance of one indicator over another; 2, 4, 6, and 8 represent intermediate judgments. Reciprocal values were assigned to the corresponding inverse comparisons. The resulting judgment matrices were normalized using the sum-normalization (Sigma) method. Specifically, each element of a judgment matrix was divided by the sum of its corresponding column, and the indicator weights were obtained by averaging the normalized values in each row. The complete pairwise comparison matrices and weight-calculation procedure are provided in Supplementary Table S2.

**Table 6:** Indicator weight values.

A	B1	B2	C1	C2	C3	C4	C5	C6	CR
1	0.50	0.50	0.17	0.53	0.22	0.08	0.25	0.75	<0.1

A, B1, B2, and C1–C6 denote the evaluation indicators. CR represents the consistency ratio of the judgment matrix.

The consistency of each judgment matrix was examined using the consistency index (CI) and consistency ratio (CR). Specifically, CI was calculated from the maximum eigenvalue of the judgment matrix using Equation 3:


$$\begin{array}{l} CI=\frac{\lambda_{\max}-n}{n-1}, \end{array}$$ (3)

where λ_max_ is the maximum eigenvalue of the judgment matrix and *n* is the matrix order. The consistency ratio was then calculated using Equation 4:


$$\begin{array}{l} CR=\frac{CI}{RI}, \end{array}$$ (4)

where *RI* denotes the random consistency index corresponding to *n*. A judgment matrix was considered to have acceptable consistency when *CR* < 0.10. All judgment matrices satisfied this criterion, and the detailed consistency results are reported in Supplementary Table S3.

Based on this indicator system, both the original emergency plan texts and the emergency plans generated by the proposed method are evaluated. Among these indicators, task workflow (C5) and operational feasibility (C6) are further quantified using explicit mathematical formulations, which are detailed below.

The calculation formulas for task workflow (C5) and operability (C6) are defined using Equations 5–7:


$$\begin{array}{l} R_{i}=\frac{k_{i}}{N-1} \end{array}$$ (5)

$$\begin{array}{l} R=\frac{1}{N}\overset{N}{\underset{i=1}{\sum }}R_{i}=\frac{1}{N}\overset{N}{\underset{i=1}{\sum }}\frac{k_{i}}{N-1}=\frac{1}{N\left(N-1\right)}\overset{N}{\underset{i=1}{\sum }}k_{i} \end{array}$$ (6)

$$\begin{array}{l} O=\frac{\sum_{i=1}^{12}V_{i,3}}{\sum_{i=1}^{12}\left(V_{i,1}+V_{i,2}+V_{i,3}\right)} \end{array}$$ (7)

where C5 (task workflow) is quantified by the indicator *R*, and C6 (operability) is quantified by the indicator *O*. *N* denotes the number of operational-level tasks. The task network is not assumed to be fully or strongly connected; for each task *i*, *k*_*i*_ denotes the number of distinct other tasks that are reachable from *i* through one or more task-relation paths, while tasks in unreachable components are not counted. *V*_*i, j*_ denotes the number of verbs at task level *j* under emergency theme *i*, where *j* = 1, 2, 3 correspond to the target, intermediate, and operational levels, respectively.

For each operational-level task *i*, *R*_*i*_ represents the proportion of the other *N*−1 operational-level tasks that are reachable from task *i* through the task network. The overall workflow indicator *R* is the average of *R*_*i*_ across all operational-level tasks. Therefore, a larger *R* indicates that a greater proportion of tasks are connected through task paths, reflecting stronger workflow connectivity within the emergency plan. The operability indicator *O* represents the proportion of operational-level verbs among all target-, intermediate-, and operational-level verbs across the 12 emergency themes. A larger *O* therefore indicates that a greater proportion of plan content is represented as concrete operational actions rather than remaining at the target or intermediate level.

### 4.3 Effectiveness assessment results

Using Village A as the case study, the original emergency plan implemented in Village A was used as an empirical baseline for evaluating the proposed framework. The original emergency plan and the emergency plan generated using the HTN-Verb framework were evaluated using the same indicator definitions and AHP-derived weights, thereby enabling a direct comparison of plan adequacy and operability under a common evaluation protocol. Following the indicator system constructed in Section 4.2, relevant indicators for both types of texts were quantitatively computed, and the results are summarized in Table 7.

**Table 7:** Indicator values for two types of emergency text on major infectious diseases in Village A.

Indicator	Original emergency plan (empirical baseline)	HTN-Verb-generated plan
*C*_1_	7	12
*C*_2_	98	201
*C*_3_	12	21
*C*_4_	5	7
*C*_5_	0.26	0.44
*C*_6_	19.38%	31.34%

*C*_1_–*C*_4_ correspond to plan adequacy indicators, while *C*_5_ and *C*_6_ denote plan operability indicators.

The results show that the original emergency plan text achieves an overall effectiveness score of 0.27, whereas the generated emergency plan based on the HTN-Verb framework emergency plan attains a score of 0.73, indicating a substantial difference in effectiveness. This comparison suggests that, relative to the original plan, the HTN-Verb based emergency plan demonstrates a markedly higher overall level in terms of content completeness and task organization executability.

A further comparison across evaluation dimensions reveals that the generated plan exhibits clear improvements in both plan adequacy and plan operability, with particularly pronounced advantages in operational-level task representation and task workflow organization. These findings indicate that transforming emergency tasks from static textual descriptions into a structured and executable task network contributes to improving the overall effectiveness of emergency plans.

## 5 Conclusion and discussion

### 5.1 Implications for emergency management systems

The findings of this study offer direct managerial implications for the formulation and implementation of public health emergency plans. Analyses of emergency documents across administrative levels, real-world response texts, and the evaluation results from the Village A case reveal that existing emergency plans commonly suffer from high content homogeneity, abstract task descriptions, and an insufficient specification of operational-level tasks, which limits their effectiveness in guiding concrete actions during implementation.

In contrast, decomposing emergency response activities into a verb-centered, hierarchical task network, while explicitly specifying executing agents, task relations, and resource requirements, helps transform emergency plans from lists of responsibilities into executable task structures. This suggests that emergency management authorities, when revising emergency plans, should move beyond the practice of mechanically replicating higher-level documents and instead place greater emphasis on the systematic organization of operational-level tasks. Ensuring that key actions under different emergency themes have clear execution pathways and coordination mechanisms is critical.

Moreover, dynamically incorporating newly emerging tasks identified during actual response processes into the task verb lexicon and task relationship structures can continuously enhance the operability and adaptability of emergency plans, thereby improving the overall execution efficiency of grassroots emergency responses.

### 5.2 Comparison with existing studies

Existing studies on emergency plan generation and emergency decision support have mainly focused on case-based plan recommendation (37), predefined plan selection (38), process optimization, and resource scheduling (39). In these approaches, emergency plans are generally treated as relatively holistic and static response units, with the primary objective of retrieving similar plans, optimizing response procedures, or allocating emergency resources under predefined conditions (40). Such studies have contributed to improving decision efficiency and supporting emergency management practices.

In recent years, natural language processing techniques have also been increasingly applied to emergency management tasks (41), including emergency information extraction (42), event detection (43), and task identification from unstructured texts (44). However, many existing studies still focus primarily on information recognition or process-oriented modeling, while providing relatively limited support for the fine-grained organization of executable emergency tasks and dynamic task coordination (45).

Compared with existing studies, the proposed HTN-Verb framework emphasizes executable task construction through verb-centered semantic extraction and hierarchical task decomposition. Rather than focusing solely on plan retrieval or information recognition, the framework transforms emergency-response texts into dynamically organized task networks that support operational-level emergency planning. By integrating logical, temporal, and functional task relations, the proposed approach provides a more structured representation of task coordination and inter-task dependencies. In addition, the incorporation of real emergency-response records enables the framework to capture operational actions that are often absent from formal emergency plans, thereby improving the adaptability and practical interpretability of the generated task structures in grassroots emergency-response scenarios.

### 5.3 Conclusion

To address persistent challenges in public health emergency plans, including high content homogeneity and limited operability, this study proposes a task-oriented HTN-Verb based emergency plan generation approach that transforms static plan texts into structured and executable task networks. Centered on emergency-response verbs, the proposed HTN-Verb framework enables hierarchical task decomposition driven by verb semantics, providing a systematic pathway for extracting and organizing emergency tasks from unstructured texts. By integrating formal public health emergency plan documents with records from actual response processes, the proposed dynamic generation mechanism supports continuous enrichment and updating task structures, thereby improving alignment with real-world response demands. In addition, a multi-dimensional task relationship model encompassing logical, temporal, and functional relations is constructed, together with a priority-based conflict resolution rule, to enhance the internal consistency and executability of task networks. Evaluation results based on the Village A case demonstrate that, compared with the original public health emergency plan text, emergency plans generated using the proposed approach achieve substantially higher levels of overall effectiveness and operability.

### 5.4 Limitations

While the proposed framework demonstrates promising effectiveness in emergency plan generation and task organization, several limitations should be acknowledged.

First, the empirical evaluation is based on a single case, and the findings may be influenced by context-specific factors such as local governance, response capacity, and coordination mechanisms. Therefore, the Village A results should be interpreted as case-based evidence of framework applicability rather than as evidence of generalizability across emergency management settings. In addition, the response corpus relies on official Weibo posts and may be subject to platform-specific and institutional reporting biases, potentially underrepresenting informal or offline frontline activities. Moreover, the text-derived operational tasks may not fully capture context-specific constraints, tacit knowledge, informal coordination, and workflow adaptations encountered by frontline practitioners, as direct field-based validation with grassroots responders was beyond the scope of this study.

Second, the current framework does not achieve full automation, particularly in the extraction and modeling of task relationships. While the extraction of task elements is largely automated, the identification of relationships among tasks still requires manual verification or additional analysis. In addition, these relationship assignments have not yet been independently validated against expert annotations or annotated ground-truth data. This may limit the scalability of the framework when applied to large-scale datasets or real-time emergency scenarios.

Third, although the assignment of verbs to emergency themes was based on predefined criteria and supplemented by inter-rater validation and cross-checking, semantic ambiguity in context-dependent verbs cannot be completely eliminated. Such ambiguity may lead to variations in thematic assignments across different datasets or application contexts.

Finally, the evaluation in this study focuses primarily on the structural and semantic validity of the generated task models. Although the original Village A emergency plan was used as an empirical baseline, no directly comparable computational method was included for benchmarking, and the practical effectiveness of the generated plans has not yet been systematically evaluated in real-world or simulated environments.

### 5.5 Future work

To address the above limitations and further enhance the proposed framework, several directions for future research are identified.

The empirical validation of the framework can be extended by incorporating multiple case studies from different regions and administrative levels, provided that more detailed and comprehensive emergency response records become available. This would enable a more systematic assessment of the framework's generalizability, robustness, and adaptability across diverse emergency management contexts.

The level of automation can be further improved by incorporating advanced relation extraction techniques, such as joint entity-relation extraction models. These approaches enable more accurate and scalable identification of relationships among tasks, including interactions between actions, agents, and resources, thereby reducing the need for manual verification and enhancing practical applicability in large-scale and dynamic scenarios.

The rule-based verb-theme classification can be enhanced by adopting supervised or semi-supervised learning approaches trained on annotated emergency response datasets. Such methods can improve the robustness and consistency of task classification while reducing reliance on manually defined rules.

More comprehensive evaluation strategies can also be introduced, including simulation-based experiments and scenario-driven testing. These approaches would support a more thorough assessment of the operational effectiveness of the generated emergency plans and their performance in realistic emergency response settings. Such future validation could also involve frontline public health practitioners through interviews, participatory workshops, or scenario-based field testing to refine the task structures and assess their practical usability before deployment.

## Data Availability

The original contributions presented in the study are included in the article/Supplementary material, further inquiries can be directed to the corresponding author.
