# Supplementary material for: A task-oriented framework for dynamic generation of public health emergency plans in major infectious disease response
Source: Front Public Health. 2026 Oct 27;14:1831748. doi: 10.3389/fpubh.2026.1831748 (PMC13748333; doi:10.3389/fpubh.2026.1831748)
Supplement: Supplementary file 1 [file Data_Sheet_1.pdf]

Supplementary Material

1 SUPPLEMENTARY TABLES

Table S1. Computational implementation and parameter settings.

Component	Setting
Programming language	Python 3.12.3
Chinese text processing	THULAC 0.2.2
Pretrained language model	bert-base-chinese
Transformers	4.46.1
PyTorch	2.5.1+cu118
BERT embedding dimension	768
Pooling strategy	Average pooling
Embedding batch size	8
Dimensionality reduction	PCA
PCA components	2
PCA random state	42
Clustering algorithm	Agglomerative Clustering
Distance metric	Euclidean
Linkage criterion	Ward
Number of clusters	3
Clustering implementation	scikit-learn 1.4.2
Cluster validation	Silhouette coefficient

Table S2. Pairwise comparison matrices used for AHP weighting.

(a) First-level criteria

	Plan adequacy	Plan operability
Plan adequacy	1	1
Plan operability	1	1

(b) Secondary indicators of plan adequacy

	Theme	Task	Agent	Resource
Theme	1	1/3	1/2	3
Task	3	1	4	5
Agent	2	1/4	1	3
Resource	1/3	1/5	1/3	1

(c) Secondary indicators of plan operability

	Task workflow	Operational feasibility
Task workflow	1	1/3
Operational feasibility	3	1

Table S3. Consistency assessment of the AHP judgment matrices.

Judgment matrix	$\lambda_{\max}$	CI	CR
First-level criteria	2.0000	0	0
Plan adequacy indicators	4.1621	0.0540	0.0600
Plan operability indicators	2.0000	0	0
